# ArchiLD: Hierarchical Visualization of Linkage Disequilibrium in Human Populations

**DOI:** 10.1371/journal.pone.0086761

**Published:** 2014-01-21

**Authors:** Rossella Melchiotti, Olaf Rötzschke, Michael Poidinger

**Affiliations:** 1 Singapore Immunology Network (SIgN), Agency for Science, Technology and Research (A*STAR), Singapore, Singapore; 2 Doctoral School in Translational and Molecular Medicine (DIMET), University of Milano-Bicocca, Milan, Italy; Seoul National University College of Medicine, Republic of Korea

## Abstract

Linkage disequilibrium (LD) is an essential metric for selecting single-nucleotide polymorphisms (SNPs) to use in genetic studies and identifying causal variants from significant tag SNPs. The explosion in the number of polymorphisms that can now be genotyped by commercial arrays makes the interpretation of triangular correlation plots, commonly used for visualizing LD, extremely difficult in particular when large genomics regions need to be considered or when SNPs in perfect LD are not adjacent but scattered across a genomic region. We developed ArchiLD, a user-friendly graphical application for the hierarchical visualization of LD in human populations. The software provides a powerful framework for analyzing LD patterns with a particular focus on blocks of SNPs in perfect linkage as defined by r^2^. Thanks to its integration with the UCSC Genome Browser, LD plots can be easily overlapped with additional data on regulation, conservation and expression. ArchiLD is an intuitive solution for the visualization of LD across large or highly polymorphic genomic regions. Its ease of use and its integration with the UCSC Genome Browser annotation potential facilitates the interpretation of association results and enables a more informed selection of tag SNPs for genetic studies.

## Introduction

The development and diffusion of high-throughput technologies for the analysis of genetic variants, such as single-nucleotide polymorphism (SNP) microarrays and next-generation sequencing, has lead to a substantial increase in the number of variants that can be included in population-based genetic studies [1]. Commercially available microarray platforms can now measure up to 5 million SNPs on a single chip.

A large number of these variants are not independent but correlated through linkage disequilibrium (LD), the non-random association of alleles at two genomic locations. Knowledge of LD plays an important role in the selection of SNPs to be tested for association with a particular phenotype. This metric can in fact be used to alleviate the burden of multiple testing by pruning out redundant information. It is also useful for identifying possible causative variants from relevant tag polymorphisms.

The completion of the International HapMap project [2]–[5] and the 1000 Genomes Pilot Project [6] has provided scientists with a high-density map of genetic variation across the major human populations making it possible to evaluate patterns of LD on a genome-wide scale.

Triangular correlation plots for the visualization of pairwise linkage disequilibrium across genome regions, as implemented in Haploview [7], are the most used method to report LD information in population-based genetic studies [8]. However, the extensive number of variants now available on commercial arrays makes the interpretation of triangular correlation plot difficult especially when SNPs in high linkage are not adjacent but interspersed between other SNPs. Furthermore the complexity of triangular correlation plots increases with the size of the region of interest which complicates the analysis of LD patterns across large genes. It is therefore necessary to develop alternative ways to plot LD for genome regions characterized by a high density of SNPs or fragmented LD blocks.

We conceived a new way of visualizing LD patterns across the genome as blocks of SNPs in perfect LD (r^2^ = 1) that can be hierarchically clustered based on their pairwise linkage. ArchiLD is the implementation of this new concept in a user-friendly Java application which integrates the UCSC Genome Browser [9] visualization potential with a simple and intuitive tool for building LD blocks.

### Implementation

ArchiLD comes in two versions: a client-server application (ArchiLD1k) for the analysis of LD across the four populations sequenced by the 1000 Genomes Pilot Project (CEU, CHB, JPT and YRI) [6] and a standalone application (ArchiLDCustom) for the analysis of LD across custom genotypic datasets.

ArchiLD1k computes blocks of SNPs in perfect LD (r^2^ = 1), called clusters, from pre-calculated pairwise LD measures obtained using the software Haploview [7] on the four populations provided by the 1000 Genomes Pilot Project [6]. Due to the low number of CHB and JPT samples sequenced by the consortium (30 samples each) when compared with the number of YRI and CEU samples (respectively 59 and 60), the two Asian populations have been merged together as previously done by others [6], [10]. This allows for an easier comparison of LD patterns across populations due to their similar sample size. The software will soon be updated to include all 1092 individuals (14 distinct populations) sequenced by the 1000 Genome Project phase I [11]. More individuals/populations will be added as they become available.

ArchiLDCustom computes LD clusters from custom genotyping datasets imported by the user. The software accepts pedigree data and marker information in the standard linkage format used by Haploview [7]. Each chromosome needs to be loaded independently and r^2^ values are estimated using the software Haploview [7].

In both versions of the software clusters are visualized as custom tracks in an integrated instance of the UCSC Genome Browser [9].

ArchiLD1k is implemented as a client-server application developed in Java v1.6 (client-side) and Java EE v1.7 (server-side). All computations are carried out by a Java servlet deployed on Apache Tomcat (v7.0.30). Hierarchical clustering of LD blocks is performed using R v2.15.1 by the function hclust from the package {stats} utilizing an agglomeration method based on average [12]. The distance matrix used for the analysis is defined from LD pairwise measures as 1-r^2^. Analysis parameters can be selected by means of a query interface on the client side and are then dispatched to the servlet which computes LD blocks and generates the custom Browser Extensible Data (BED) tracks used for plotting clusters. BED is a file format used by the UCSC Genome Browser [9] to define genomic regions: a description of the required fields can be found on the UCSC Genome Bioinformatics website [13]. Custom tracks are automatically imported in the browser when the user selects which architecture to visualize. Gene annotations were downloaded from the UCSC Genome Browser database [14] and SNP annotations were provided by the 1000 Genome Pilot Project (genome build hg18) [6]. Hg19 positions were obtained using the liftOver tool provided by the UCSC Genome Browser [15]. Minor allele frequencies were computed using the software PLINK [16] on all samples sequenced by the 1000 Genomes Pilot Project (60 CEU, 60 CHB+JPT and 59 YRI) [6]. Each population was analyzed independently. Tables containing LD information and gene/SNP annotations are managed on the server side using MySQL v 5.5.27.

ArchiLDCustom is completely implemented in Java v1.6. Hierarchical clustering of LD blocks is performed by a local instance of R using the same algorithm described for ArchiLD1k. Annotation tables and custom datasets are managed through a MySQL database. Connection parameters to the MySQL server and the complete path to the local installation of Haploview and R need to be set before any analysis can be performed. All the computations are run locally. LD plots can be visualized using an integrated instance of the UCSC Genome Browser [9] but contrarily to the client-server version tracks containing LD plots need to be uploaded manually in the browser. All plots are stored as BED files.

All graphical interfaces were implemented using the Qt libraries for Java [17].

## Results

### LD architectures

LD architectures, sets of clusters of SNPs in perfect LD (r^2^ = 1), can be built starting from four distinct genomic elements: genes, SNPs, chromosomes (ArchiLD1k only) and genomic regions (ArchiLDCustom only).

Gene-centered architectures are composed of LD blocks with at least one SNP located inside a gene or in proximity of it. The maximum distance between the gene transcription start/end and the SNPs to be included in the analysis can be adjusted by the user. The tool accepts three different identifiers for gene names: Entrez IDs, RefSeq IDs and HUGO gene symbols.

SNP-centered architectures contain all clusters in LD with a selected SNP (reference SNP). To reduce the size of the tables used for the computations and the processing time required to generate the clusters only variants with an r^2^> = 0.5 with the reference SNP are considered.

Chromosome-centered architectures contain all LD blocks located on a specific chromosome. Region-centered architectures focus on the genomic region described in the genotypic dataset imported by the user. Only SNPs included in the file are used to build clusters.

Regardless of the type of analysis selected, users can set a minor allele frequency threshold to exclude rare SNPs from the analysis and choose which genome build (hg18/hg19) to use for the visualization (ArchiLD1k only).

### Visualization

LD architectures are visualized as custom BED files in the integrated instance of the UCSC Genome Browser [9]. SNPs in perfect LD (r^2^ = 1) are joined by a horizontal line which constitutes a cluster.

**Figure 1:**
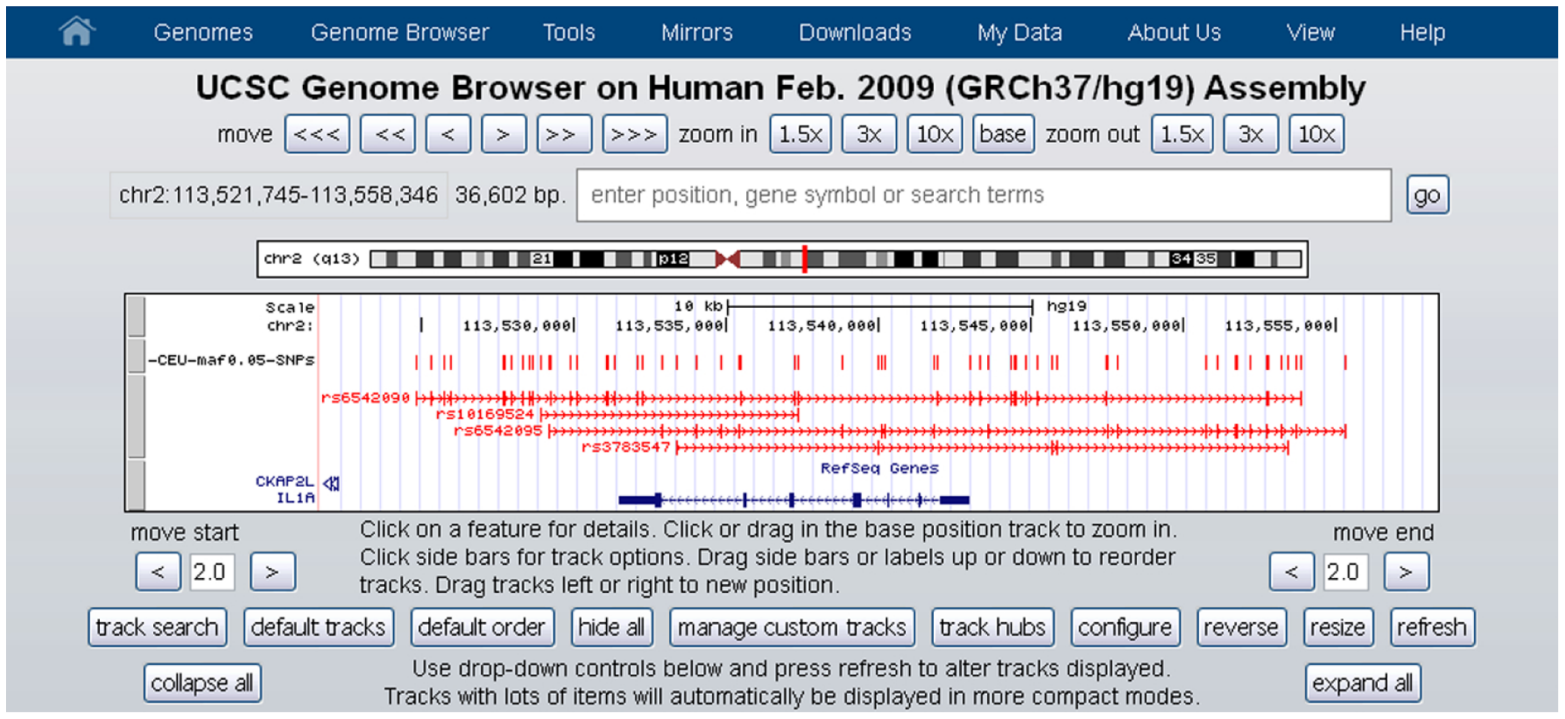
An example of gene-centered architecture. The first track contains the names and positions of all SNPs considered in the analysis. The second track contains all clusters associated to the gene.

Gene-centered architectures are represented by two distinct tracks, one containing the name and position of individual SNPs and one containing all blocks in the region. Clusters are identified by the name of their first SNP (Figure 1). Users can decide to include only SNPs belonging to a cluster or all the SNPs in the region. If SNPs not belonging to any LD block (singletons) are included, a new track is added to the visualization.

**Figure 2:**
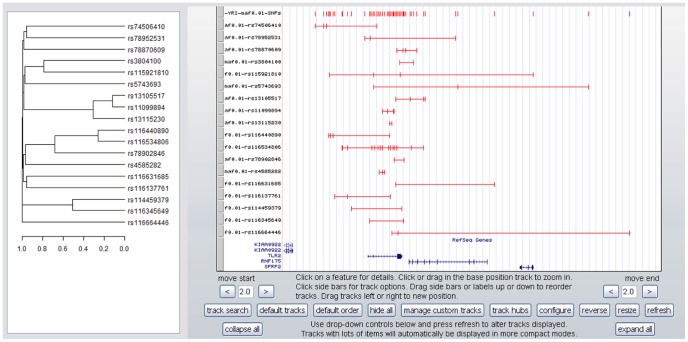
An example of hierarchical clustering. On the left the hierarchical clustering of all SNP blocks associated to a particular gene. On the right a graphical representation of a gene-centered architecture. The first track contains the names and positions of all SNPs considered in the analysis. The following tracks are ordered as they appear in the hierarchical clustering plot.

It is also possible to build a hierarchical tree of clusters spanning the gene. Blocks of SNPs in perfect LD are clustered according to their pairwise r^2^. Hierarchical plots are displayed next to the Genome Browser window to facilitate the interpretation of LD patterns. When this option is selected each cluster is represented by a distinct track with the order of the tracks following the order of the clusters in the hierarchical tree (Figure 2).

Region-centered architectures are similarly visualized but only SNPs included in a custom genotypic dataset are used for the analysis.

**Figure 3:**
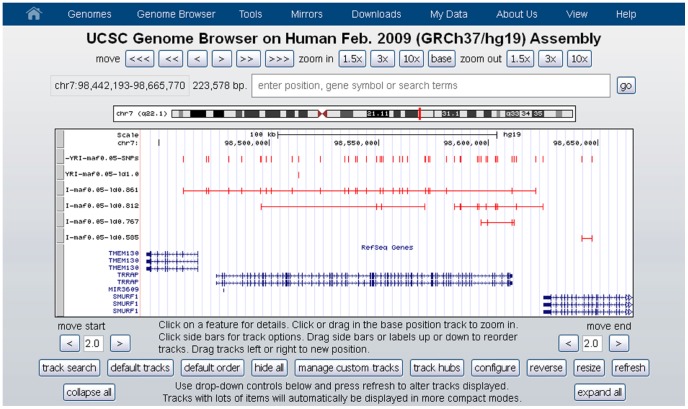
An example of SNP-centered architecture. The first track contains the names and positions of all SNPs considered in the analysis. The second track contains the reference SNP and its linked variants. The following tracks are ordered by descending r^2^ with respect to the reference SNP.

SNP-centered architectures are represented by multiple tracks. The first track contains SNP names. The second track displays the reference SNP and all its perfectly linked SNPs. All other tracks are ordered by decreasing r^2^ values (the corresponding r^2^ is included in the track name, Figure 3). Singletons can be included and added to the track with the appropriate r^2^ value.

For gene-centered, SNP-centered and region-centered architectures the user has the option to color SNPs and LD blocks by minor allele frequency. This simplifies the identification of clusters of SNPs in linkage disequilibrium with similar allele distributions.

**Figure 4:**
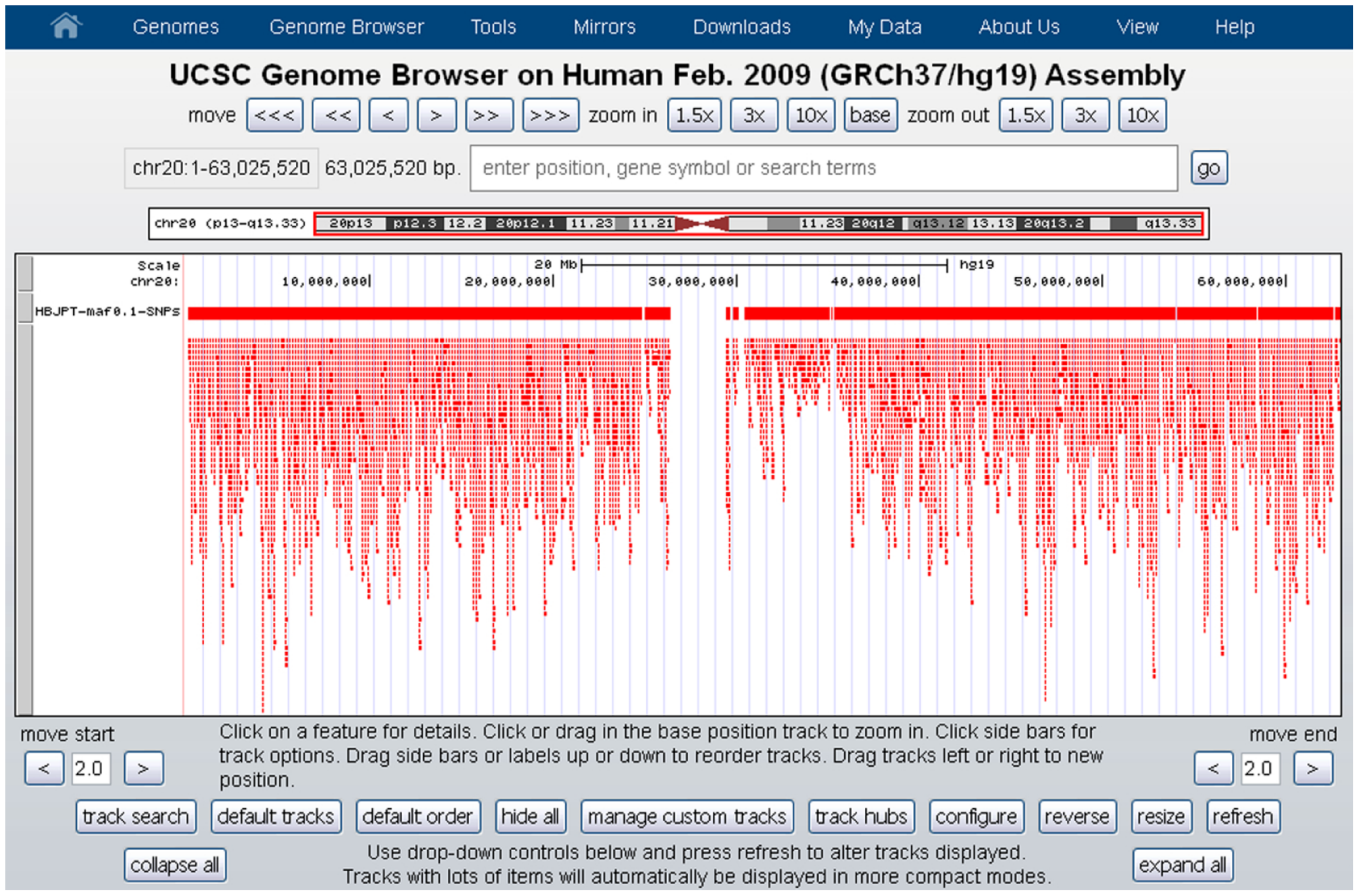
An example of chromosome-centered architecture. The first track contains the names and positions of all SNPs considered in the analysis. The second track contains all clusters located on the chromosome.

For chromosome-centered architectures all clusters are added to the same track (Figure 4). When singletons are included an additional track containing only these SNPs is added to the visualization.

BED tracks and filenames containing the IDs of SNPs in a particular cluster can be easily exported for external use using the navigation tree. Multiple plots can be uploaded simultaneously as UCSC tracks to facilitate the comparison of LD patterns across different populations.

In ArchiLD1k, gene-centered and SNP-centered architectures can be automatically loaded into the browser by clicking on the corresponding item in the navigation tree. Due to their large size chromosome-centered architectures cannot be automatically loaded but need to be exported first and then manually imported. ArchiLDCustom requires users to export BED tracks and manually import them in the browser regardless of the type of architecture generated.

## Discussion

ArchiLD is a powerful software for producing highly interpretable plots that can be used to select tag SNPs in the context of association studies or to prioritize SNPs for functional studies. Its integration with the UCSC Genome Browser [9] makes it easy to overlap additional information about regulation, conservation across species, phenotype and disease associations aiding the users in the interpretation of their results.

### Query performance

Generation of SNP-centered architectures requires few seconds. For gene-centered architectures the process can take from few seconds to several minutes according to the length of the gene and the size of the upstream/downstream region selected by the user. Chromosome-centered architectures are pre-computed but due to their large size require minutes to be uploaded into the UCSC Genome Browser [9]. The import of custom datasets can be time-consuming depending on the number of SNPs included in the file: the limiting factor is the computational time required by Haploview [7] to generate pairwise LD measures.

### Comparison with similar software

**Figure 5:**
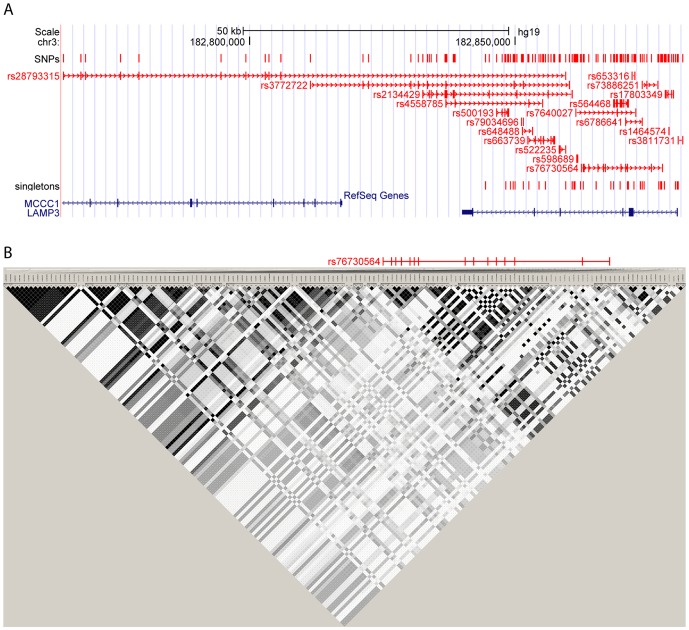
Comparison between ArchiLD and Haploview. (A) Gene-centered visualization for the gene LAMP3 in CEU as provided by ArchiLD1k. (B) Analogous visualization in Haploview. The large number of variants analyzed makes it difficult, for example, to identify the position of the SNPs in perfect LD with rs76730564.

Multiple tools have so far been developed to tackle the complexity of linkage disequilibrium visualization in human populations. Haploview [7] is one of the most used software for the computation and visualization of LD. It is extremely powerful for visualizing small genomic regions where highly linked SNPs are organized in compact blocks and it is therefore strongly used for tag selection in candidate gene studies. Triangular correlation plots become difficult to interpret when the region of interest contains a large number of SNPs or when highly linked SNPs are not adjacent (Figure 5). ArchiLD facilitates the analysis of LD across these regions by joining perfectly linked SNPs in visual clusters and by ordering clusters with respect to their relative LD: SNP-centered architectures are ordered by decreasing r^2^ with respect to a reference SNP (index SNP) while gene-centered and region-centered architectures are ordered according to their hierarchical tree with strongly linked blocks clustered together. In Haploview LD computations are done on the fly, a time and memory consuming process. ArchiLD1k on the other hand uses pre-computed r^2^ values for building and hierarchically organizing LD blocks and can thus be used on a very large scale.

Another interesting way of computing LD clusters is offered by LD-based clumping, a technique implemented, for example, in PLINK [16]. The clumping procedure is straightforward: it requires a SNP dataset and an input file containing variant names and association p-values. SNPs with an assigned p-value lower than a certain threshold (user-based) are taken as index SNPs. All the other SNPs in the region are assigned to their most closely linked index SNP. An r^2^ threshold can be selected to ignore variants that are weakly linked to index SNPs. The output of the procedure is a text file containing the list of index SNPs (with their respective association p-values) and the list of their linked SNPs. The information provided by the clumping procedure is extremely valuable for the interpretation of association results and the selection of genetic variants to use in downstream analysis but the visualization of the results is not straightforward. ArchiLD does not offer any clumping functionality since our visualization of linkage disequilibrium is independent of the availability of association results. Nonetheless clumping could be performed in two steps: by manually analyzing an association file to identify index SNPs and by creating a SNP-centered architecture for each index SNP.

**Figure 6:**
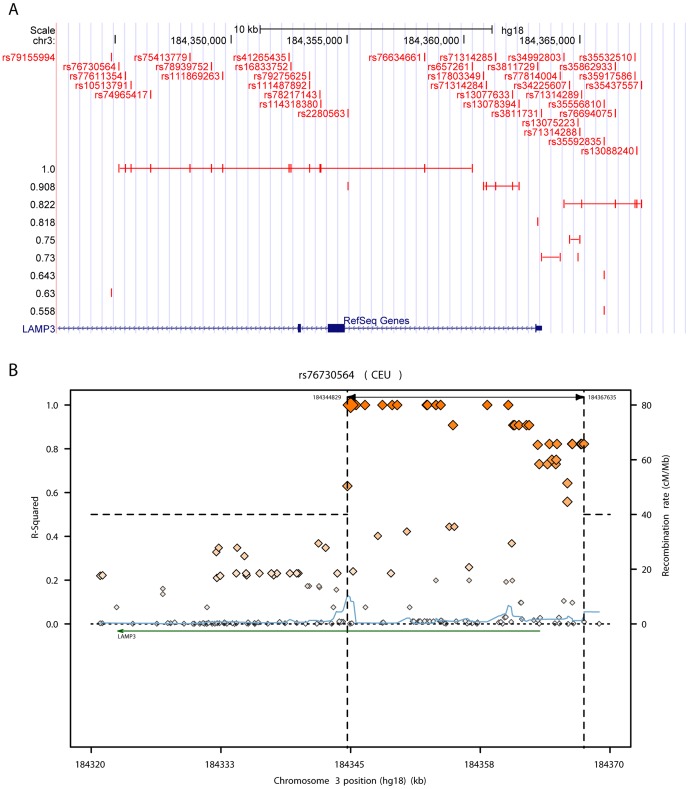
Comparison between ArchiLD and SNAP. (A) SNP-centered visualization for the variant rs76730564 in CEU as provided by ArchiLD1k. Clusters are ordered by descending values of r^2^ with the reference SNP. (B) Analogous visualization in SNAP. The plot does not contain any SNP label nor provide any information on the location of exons.

Another well used software for the analysis of LD patterns across the genome is SNAP [10]. The plots produced by SNAP are very similar to the SNP-centered plots produced by ArchiLD but our software has the advantage of including individual SNP labels and visualizing the gene structure (number of transcripts, positions of the exons) (Figure 6). SNAP can also be used to integrate association results with LD information. The LD plot is by default centered on the SNP with the highest association signal (this can be modified by the user). The tool does not offer any way of focusing on a particular gene/region. This limitation is overcome by other tools such as LocusZoom [18] where the user can choose a gene or a genomic region to analyze. As for SNAP the plot is centered on the SNP with the lowest p-value but this can be easily modified by the user. Both HapMap [2]–[5] and the 1000 Genome Project datasets [6], [11] can be used for computing LD. The advantage of this tool with respect to ArchiLD lies in the availability of pre-loaded GWAS datasets. Custom association datasets can also be loaded for the visualization. The integration of LD plots with pre-loaded GWAS datasets is also offered by Ricopili, a tool developed by the Broad Institute [19]. As for LocusZoom the association/LD plot can be centered on a particular gene or a particular genomic region. The advantage of Ricopili with respect to LocusZoom is that more than one index SNP can be used for the visualization (clumping). When more than one reference variant is selected the most associated SNPs are chosen as index SNPs and all the other SNPs are assigned to their most closely linked reference SNP. SNPs are colored according to their pairwise LD with the reference SNP they are assigned to. The main disadvantage of this kind of visualization is that it does not provide any information about the pairwise LD of SNPs with the same color. It is impossible to say from the plot if these SNPs are totally independent or are strongly linked. ArchiLD tackles this limitation by joining SNPs which are perfectly linked with a horizontal line. While ArchiLD does not provide any pre-loaded GWAS dataset, custom association p-values can be imported as bedGraph custom tracks [13] and easily overlapped with the LD plot in the integrated browser. Except for Haploview all of the tools mentioned above generate SNP-centered plots: the plot can be centered on a particular gene but one or more reference SNPs need to be selected before a visualization can be created. ArchiLD not only provides options to generate gene-centered LD patterns but also offers the possibility of clustering the resulting LD blocks using a hierarchical tree. A strong advantage of ArchiLD over the aforementioned tools is that it does not produce static pdf plots but interactive plots thanks to its integration with the UCSC Genome Browser [9]. Plots can be modified in real-time by zooming in/out or shifting the visualization upstream/downstream. Once the user is satisfied with the visualization a pdf file can be created.

The major drawback of all the tools here described when compared with ArchiLD consists in the difficulty of adding functional annotations to LD plots (besides association p-values or in the case of Ricopili NHGRI GWAS catalog variants) : ArchiLD integration with the UCSC Genome Browser [9] provides a solution to this limitation thanks to the large number of free annotation tracks available.

### Availability

Precompiled binaries for both ArchiLD1k and ArchiLDCustom (Windows, Linux and Mac OS) can be downloaded from the project website (http://archild.sign.a-star.edu.sg) [20]. ArchiLD1k binaries are distributed as zip files containing a runnable jar and all the libraries required by the application. ArchiLDCustom binaries are distributed as zip files containing a runnable jar, all the libraries and annotation tables required by the application and two sample datasets that can be used to test the software. The two sample datasets have been generated using genotype data from the 1000 Genomes Pilot Project [6] for the first 500 kbp of chromosome 7 (CEU) and chromosome 12 (CHB+JPT) respectively. ArchiLDCustom requires R v 2.15.1 and access to a MySQL database (MySQL v 5.5.27 or higher).

Instructions on how to launch the software under different operating systems and how to use the different functionalities of ArchiLD are described in the manual, available for download on the project website. Source files can also be obtained from the website and easily imported as java projects in Eclipse IDE for Java Developers. The software is released under the GNU General Public License (GPL) version 3.

## Conclusions

ArchiLD is a user-friendly application for the visualization of linkage disequilibrium in human populations. The software was developed to aid geneticists in selecting SNPs to include in genetic studies and in identifying putative causative SNPs from relevant tag variants. Its ease of use and high interpretability make ArchiLD a powerful addition to every geneticist's toolbox.
